# MoS_2_ Nanosheet Arrays Rooted on Hollow rGO Spheres as Bifunctional Hydrogen Evolution Catalyst and Supercapacitor Electrode

**DOI:** 10.1007/s40820-018-0215-3

**Published:** 2018-07-09

**Authors:** Shizheng Zheng, Lijun Zheng, Zhengyou Zhu, Jian Chen, Jianli Kang, Zhulin Huang, Dachi Yang

**Affiliations:** 1 Key Laboratory of Photoelectronic Thin Film Devices and Technology of Tianjin, Department of Electronics, College of Electronic Information and Optical Engineering, Nankai University, Tianjin, 300350 People’s Republic of China; 2 School of Material Science and Engineering, Tianjin Polytechnic University, Tianjin, 300387 People’s Republic of China; 3 Key Laboratory of Materials Physics, and Anhui Key Laboratory of Nanomaterials and Nanotechnology, Institute of Solid State Physics, Chinese Academy of Sciences, Hefei, 230031 People’s Republic of China

**Keywords:** MoS_2_, Reduced graphene oxide (rGO), Hollow spheres, Hydrogen evolution reaction (HER), Supercapacitor

## Abstract

MoS_2_ has attracted attention as a promising hydrogen evolution reaction (HER) catalyst and a supercapacitor electrode material. However, its catalytic activity and capacitive performance are still hindered by its aggregation and poor intrinsic conductivity. Here, hollow rGO sphere-supported ultrathin MoS_2_ nanosheet arrays (h-rGO@MoS_2_) are constructed via a dual-template approach and employed as bifunctional HER catalyst and supercapacitor electrode material. Because of the expanded interlayer spacing in MoS_2_ nanosheets and more exposed electroactive S–Mo–S edges, the constructed h-rGO@MoS_2_ architectures exhibit enhanced HER performance. Furthermore, benefiting from the synergistic effect of the improved conductivity and boosted specific surface areas (144.9 m^2^ g^−1^, ca. 4.6-times that of pristine MoS_2_), the h-rGO@MoS_2_ architecture shows a high specific capacitance (238 F g^−1^ at a current density of 0.5 A g^−1^), excellent rate capacitance, and remarkable cycle stability. Our synthesis method may be extended to construct other vertically aligned hollow architectures, which may serve both as efficient HER catalysts and supercapacitor electrodes.

**Electronic supplementary material:**

The online version of this article (10.1007/s40820-018-0215-3) contains supplementary material, which is available to authorized users.

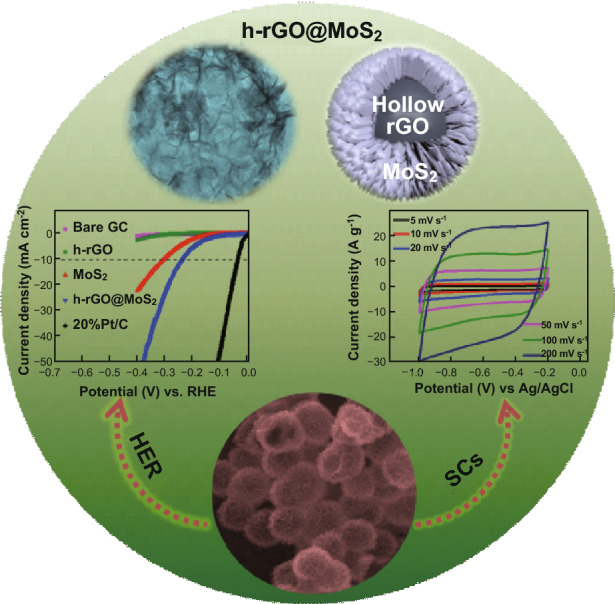

## Highlights

- MoS_2_ nanosheets arrays were vertically rooted on hollow rGO spheres (h-rGO@MoS_2_) via an optimized dual-template strategy.
- The bifunctional h-rGO@MoS_2_ architecture exhibit enhanced hydrogen evolution reaction performance (105 mV, onset potential) and higher specific capacitance (238 F g^−1^ at 0.5 A g^−1^) as a supercapacitor electrode than pristine MoS_2_.

## Introduction

The worsening energy crisis and environmental pollution have stimulated increased research into exploiting sustainable, renewable energy sources, and advanced energy-storage devices. Hydrogen, a clean energy source with the highest gravimetric energy density (143 kJ g^−1^) [1], is considered as a promising alternative to fossil fuels and has, thus, attracted significant attention. Water electrolysis is a simple way to produce highly pure hydrogen; but the best-performed Pt-based electrocatalysts suffer from their high cost and scarcity of platinum [2, 3]. Additionally, due to the low cost, rapid charge–discharge process, and long cycling stability, supercapacitors have emerged as promising energy-storage devices to meet the burgeoning demand [4, 5]. Nevertheless, the energy density of supercapacitors is still less than satisfactory [6].

To address this bottleneck, emerging 2D materials, particularly MoS_2_, have been widely studied as hydrogen evolution reaction (HER) catalysts [7–14] and supercapacitor electrode materials [15, 16]. Unfortunately, because of the poor intrinsic conductivity and easy aggregation, the HER catalytic activity and supercapacitive performance of bulk MoS_2_ are poor [17, 18]. Accordingly, significant efforts have been devoted to confine the growth of MoS_2_ using conductive matrix as template [19, 20]. Reduced graphene oxide (rGO), a layered carbon material with large-specific surface area and excellent electrical conductivity, has been proven to be an effective matrix that can endow MoS_2_ with specific shapes, expand the interlayer spacing, and increase the conductivity [17, 21–26]. For example, Dai and coworkers synthesized a MoS_2_/rGO hybrid structure via a solvothermal process, and the product benefitted from the synergistic effect of strong chemical and electronic coupling effects, an abundance of electroactive edges, and improved conductivity; thus, the hybrid exhibited enhanced HER performance [17]. Although remarkable progress has been achieved toward improving the HER performance by introducing graphene oxide as a growth matrix, the performance is still far from satisfactory. On the one hand, the inevitable restacking of graphene oxide, arising from the strong sheet-to-sheet *π*–*π* interactions [27], hampers the full use of the active surfaces. On the other hand, the limited ion diffusion and mass transport of the flat structure lead to the unsatisfactory HER catalytic activity.

Construction of hierarchical hollow architectures is deemed to be an effective strategy to enhance the electrochemical properties because of the advantages of a high surface-to-volume ratio and open structure [28–30]. In addition, the cavities in the hollow structures can serve as “ion-buffering reservoirs” to shorten the ion transport distances [31]. Previous papers [32, 33] have reported the application of conductive carbon shells as supports, yielding hierarchical hollow architectures; however, the lack of functional groups on surface constrains the growth of MoS_2_ to ultrathin nanosheets. Moreover, the relatively thick carbon shells (> 20 nm) have adverse effect on ion diffusion and mass transport. Based on the above considerations, a facile SiO_2_-template-based method is designed to fabricate hollow graphene spheres as supports to confine the growth of MoS_2_ nanosheet arrays. It is anticipated that the restacking of GO would be prevented by SiO_2_ templates, and MoS_2_ is expected to be vertically supported on the GO shells. This expected hollow architecture possesses advantages of enhanced surface area, as well as more exposed electroactive sites, and, thus, is expected to exhibit enhanced HER catalytic activity and supercapacitive performance [34]. However, there have been only few reports on the use of hollow rGO spheres as a matrix to construct hierarchical architectures that serve both as HER catalysts and supercapacitor electrode materials.

**Scheme 1:**
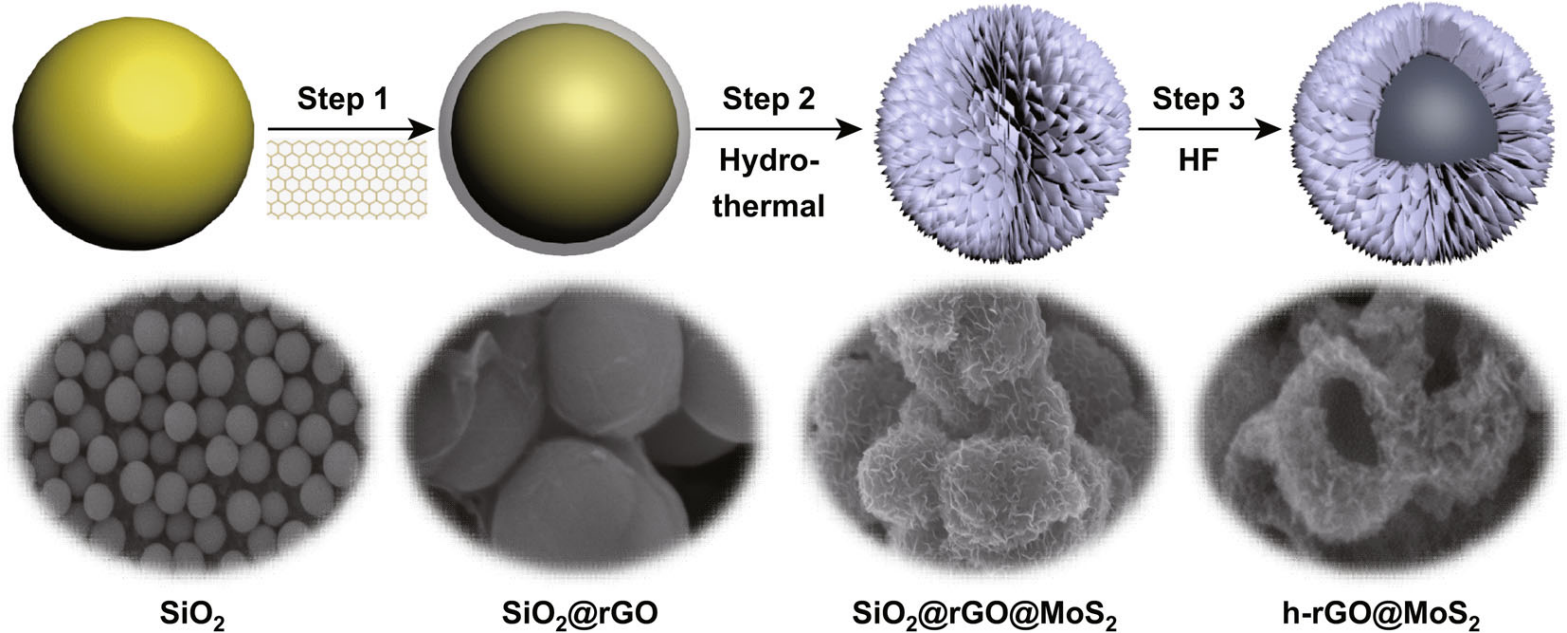
Schematic illustration of the synthetic process of h-rGO@MoS_2_

In this study, 3D rGO hollow sphere-supported ultrathin MoS_2_ nanosheets were prepared, as illustrated in Scheme 1. First, GO was intimately coated around positively charged SiO_2_ spheres by electrostatic incorporation. Subsequently, ultrathin MoS_2_ nanosheets were vertically grown around the GO shells via a hydrothermal process. Finally, the SiO_2_ cores were fully removed by chemical etching. In such 3D architectures, the vertically aligned MoS_2_ nanosheets with a large number of electroactive S–Mo–S edges and expanded (002) interlayer spacings were observed to be tightly anchored on the graphene surface. Benefitting from the expanded (002) interlayer spacing and the exposed electroactive S–Mo–S edges, the h-rGO@MoS_2_ structure exhibited efficient HER catalytic activity with an overpotential of ca. 230 mV at 10 mA cm^−2^. In addition, because of the boosted specific surface area and conductivity, remarkable supercapacitive performance with high specific capacitance (238 F g^−1^ at 0.5 A g^−1^) was achieved.

## Experimental Section

### Synthesis of the h-rGO@MoS_2_ Architecture

First, GO sheets and SiO_2_ spheres were prepared via a modified Hummers’ method [35] and typical Stöber method [36], respectively. Before the GO wrapping process, SiO_2_ spheres were decorated with (3-aminopropyl) triethoxysilane (APTES) [37] to obtain a positively charged surface. Second, during the GO coating process, 0.5 g of positively charged SiO_2_ was dispersed in 300 mL of DI-water in a supersonic bath, and, then, 50 mL of 0.1 mg L^−1^ GO was dropped into the solution with continuous stirring. Subsequently, the SiO_2_@GO powders were obtained after filtration and drying in an oven at 60 °C, followed by grinding. Next, 0.5 g of the as-synthesized SiO_2_@GO powder was re-dispersed in 35 mL DI-water, followed by the addition of 0.3 g of Na_2_MoO_4_·2H_2_O and 0.8 g of thiourea to the solution. Subsequently, 1 mL concentrated HCl (36 wt%) was added to adjust the pH value. After 1 h stirring, the blue mixture was transferred to a 50-mL Teflon-lined stainless-steel autoclave and kept at 200 °C for 24 h. After that, the black SiO_2_@rGO@MoS_2_ sample was washed thoroughly and dried at 60 °C, followed by vacuum calcination at 350 °C for 2 h. Finally, the SiO_2_ templates were removed via etching in 10% HF while stirring for 12 h, and h-rGO@MoS_2_ hollow spheres were obtained. For comparison, SiO_2_/MoS_2_ and pristine MoS_2_ were synthesized under the same conditions without the addition of GO and SiO_2_@rGO template. Additionally, hollow rGO(h-rGO) spheres were synthesized via GO coating, hydrothermal reduction, and a SiO_2_ etching process.

### Characterization

The as-prepared samples were characterized by X-ray diffraction (XRD) using a Cu Kα radiation source (*λ* = 1.5406 Å) operating at 40 kV and 100 mA. Field-emission scanning electron microscopy (FE-SEM, JEOL-6701F), transmission electron microscopy (TEM, JEOL-2010), and high-resolution TEM (HRTEM, JEOL-2010) measurements were also carried out. X-ray photoelectron spectroscopy (XPS) measurements were carried out using a Thermo Scientific ESCALAB 250Xi instrument. The specific surface areas were analyzed using a Bei Shi De (3H-2000PM2) instrument and calculated using the Brunauer–Emmett–Teller (BET) model. The Raman spectra were recorded using an RTS-HiR-AM with excitation at 532 nm and a power of 5 μW.

### Electrochemical Evaluations

For HER evaluation, the electrochemical tests were carried out in a three-electrode system with 0.5 M H_2_SO_4_ as the electrolyte and a Ag/AgCl electrode and graphite rod as the reference and counter electrodes, respectively. For these experiments, 10 mg pristine MoS_2_ and 10 mg h-rGO@MoS_2_ were dispersed in 1 mL liquor (0.75 mL water + 0.15 mL alcohol + 0.1 mL 5 wt% Nafion) and sonicated for 2 h. Then, 8 μL liquor was dropped onto the glassy carbon (GC) electrode using a microsyringe and dried at room temperature, and the mass loading of catalyst was calculated to be 400 μg cm^−2^. The electrolytes were bubbled with N_2_ before measurement and flowed over the electrolyte during the scanning process to exclude O_2_. The measured potentials were referenced to the reversible hydrogen electrode (RHE) by adding a value of (0.197 + 0.059 pH) V. All data are presented without *i*R compensation.

For the evaluation of the supercapacitor, the working electrodes were prepared similarly to previous methods [38]. First, as-prepared samples (MoS_2_ and h-rGO@MoS_2_), carbon black, and polyvinylidene difluoride (PVDF) were mixed in mass ratio of 8:1:1 and ground to slurry by adding *N*-methyl pyrrolidone (NMP). Second, the obtained slurry was then painted onto carbon cloth current collectors, yielding a calculated active area of 1 cm^2^. Finally, the resultant working electrodes were dried at 60 °C for 24 h and pressed at 10 MPa. All electrochemical experiments were performed in 1 M Na_2_SO_4_ solution with platinum foils as the counter electrode and Ag/AgCl as the reference electrode, respectively. An electrochemical work station (CH Instruments, CHI760e) was employed for the cyclic voltammetry (CV), electrochemical impedance spectroscopy (EIS) measurements, and galvanostatic charge–discharge (GCD) tests. The EIS measurements were recorded with an AC voltage amplitude of 5 mV and a frequency range from 100 kHz to 0.01 Hz. Galvanostatic charge–discharge investigations were carried out from -1 to -0.2 V at current densities of 0.5, 1, 2, 3, 4, and 6 A g^−1^.

## Results and Discussion

**Fig. 1:**
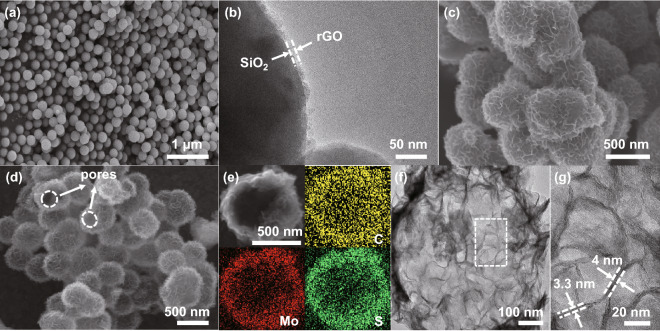
The SEM and TEM images of **a** the SiO_2_ sphere templates, **b** SiO_2_@GO, and **c** SiO_2_@GO@MoS_2_. **d** SEM images of the h-rGO@MoS_2_ spheres. **e** EDS elemental mapping results of the h-rGO@MoS_2_ spheres. C arises from rGO, Mo and S arises from MoS_2_. **f** TEM image of h-rGO@MoS_2_, and **g** the HRTEM image of the white area in **f** showing several sheets of MoS_2_

**Fig. 2:**
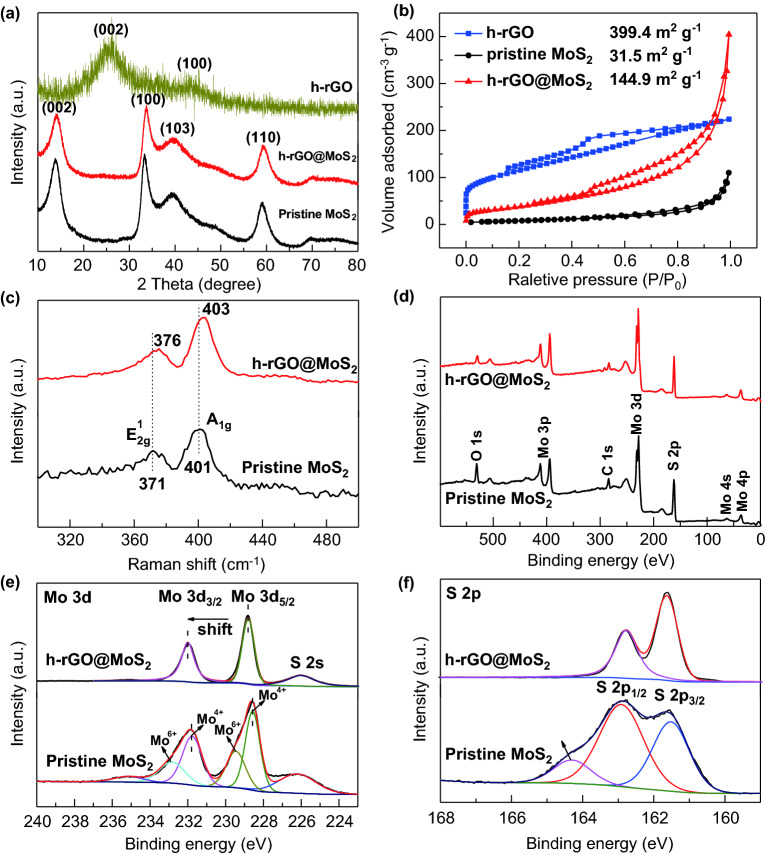
**a** XRD spectra of h-rGO, pristine MoS_2_, and h-rGO@MoS_2_. **b** N_2_ adsorption–desorption curves of h-rGO, pristine MoS_2_, and h-rGO@MoS_2_. The corresponding specific surface areas are given in the top left corner. **c** Raman spectra of pristine MoS_2_ and h-rGO@MoS_2_ measured with 532 nm excitation. **d** Survey spectra of pristine MoS_2_ and h-rGO@MoS_2_. **e**, **f** The high-resolution Mo 3d and S 2p XPS spectra of pristine MoS_2_ and h-rGO@MoS_2_

To verify the morphologies of the SiO_2_, SiO_2_@GO, and h-rGO@MoS_2_ architecture, SEM and TEM were used. Figure 1a shows an SEM image of the SiO_2_ template prepared via the Stöber method, in which the SiO_2_ particles are uniform spheres with diameters of ca. 350 nm. After decoration with APTES and coating with GO, the glossy surface of SiO_2_ was wholly wrapped with GO, the thickness of which is less than 10 nm (Fig. 1b). Accordingly, because of the template effect of the SiO_2_ spheres, the aggregation of GO sheets was hindered, and the available surface area for MoS_2_ growth was increased. As shown in Fig. 1c, the thin MoS_2_ nanosheets are vertically rooted on SiO_2_@GO surface, forming a unique 3D hierarchical architectures. This arises from the abundant hydrophilic functional groups anchored on the surface of GO, that can attract and adsorb MoO_4_^2−^ [39], resulting in the formation of well-dispersed MoS_2_ nanosheets during the hydrothermal process. Notably, none of scattered MoS_2_ nanosheets can be seen in the SEM image, revealing the appropriate addition of Mo precursors. As a comparison, SiO_2_ spheres without a GO layer were used directly as the growth template, and no MoS_2_ nanosheets were found at the roots (Fig. S1), which can be ascribed to the lack of anchoring nucleation sites for MoO_4_^2−^ on the SiO_2_ surface. Moreover, because of the stacking nature, pristine MoS_2_ sheets synthesized without SiO_2_@GO templates tended to tangle and aggregate, finally forming stacked “solid flowers” (Fig. S2). Therefore, the GO in this work serves as a template to confine the growth of the MoS_2_ nanosheets and inhibit aggregation. To achieve a hollow architecture, HF was used as the etching agent to remove the SiO_2_ template, and the final h-rGO@MoS_2_ spheres are shown in Fig. 1d. As expected, h-rGO@MoS_2_ retains its initial 3D architecture with a diameter of about 480 nm. Due to the 3D architecture, h-rGO@MoS_2_ has a large surface area and more exposed S–Mo–S electroactive edges, which was confirmed by N_2_ adsorption–desorption experiments (Fig. 2b).

To reveal the elemental distribution, energy-dispersive X-ray spectroscopy (EDS) analysis of the h-rGO@MoS_2_ was conducted, as shown in Fig. 1e. As shown, a hollow sphere with a cavity is observed, confirming the formation of a hollow architecture. Moreover, elemental C is well spatially distributed at the core, whereas Mo and S are scattered on the outer surface of the shells, indicating that MoS_2_ is well dispersed on the surface of rGO. To obtain further information about the h-rGO@MoS_2_ architecture, TEM observation was conducted, as shown in Fig. 1f. As clearly shown in the image, 3D hierarchical hollow spheres with cavities were formed, further revealing the successful formation of hollow architecture. Notably, the existence of these cavities can work as “ion-buffering reservoirs” to reduce the diffusion distance of electrolyte ions [31]. To ensure the layer structure of h-rGO@MoS_2_, corresponding HRTEM measurements are performed in Fig. 1g. As can be seen, the vertically aligned MoS_2_ nanosheets have thicknesses of about 3.3–4 nm (5–6 layers), thinner than that of pristine MoS_2_ (Fig. S2). Moreover, the interlayer spacing of the MoS_2_ nanosheets was measured to be ca. 0.65 nm (Fig. S3), which is assigned to the expanded (002) interlayer spacing of hexagonal MoS_2_ [40] and agrees well with the results of XRD analysis (Fig. S4). The expanded interlayer spacing can increase the diffusion kinetics of ions and the interlayer conductivity [41] and, thus, could enhance the HER performance. As a result, 3D hollow rGO spheres confine the growth of MoS_2_ to form highly dispersed and vertically aligned ultrathin nanosheets with large surface area, exposed S–Mo–S electroactive edges, and expanded (002) interlayer spacing, which promise remarkable HER catalytic performance and supercapacitance.

The crystallographic structures (Fig. 2a) of h-rGO, pristine MoS_2_, and h-rGO@MoS_2_ architecture were identified via XRD characterization. In the XRD analysis of rGO, two broad peaks appear at 2*θ* = 25° and 43°, demonstrating that graphene oxide was reduced to rGO. With respect to the XRD spectra of MoS_2_ and h-rGO@MoS_2_, both patterns contain peaks corresponding to semi-conductive hexagonal crystalline (2H) MoS_2_, confirming that no impurity phases were introduced via the SiO_2_ template method. It is worth noting that the (002) diffraction peak in Fig. S4 is shifted from 14.1° (MoS_2_) to 13.7° in h-rGO@MoS_2_, further revealing the expansion of (002) interlayer spacing. The broad diffraction peak corresponding to amorphous SiO_2_ (located at 2*θ* = 22.5°, Fig. S5) was not detected, confirming that the SiO_2_ templates had been successfully removed. In addition, no diffraction peaks of rGO were present on account of its low weight ratio.

The specific surface area is a key factor that has a significant influence on the HER catalytic performance and specific capacitance. To evaluate the specific surface area, N_2_ adsorption–desorption characterization was carried out for h-rGO, pristine MoS_2_, and the h-rGO@MoS_2_ architecture, and the results are shown in Fig. 2b. All the curves present type-IV hysteresis loops (IUPAC classification), which may be closely related to the flower-like MoS_2_ or 3D hollow rGO and h-rGO@MoS_2_ architectures, in accordance with the SEM observations. Because of the hollow structure and unstacked layers, h-rGO spheres exhibit the highest specific surface area of about 399.4 m^2^ g^−1^. Moreover, after the MoS_2_ nanosheets had vertically grown on the surface of h-rGO, the BET surface area of the h-rGO@MoS_2_ architecture was calculated to be 144.9 m^2^ g^−1^, which is about 4.6-times that of pristine MoS_2_ (31.5 m^2^ g^−1^). This boosted surface area originates from the confined growth of MoS_2_ and vertically aligned nanosheets, as shown in the SEM image of Fig. 1d. Moreover, the confined growth of MoS_2_ indirectly results in the exposure of electroactive S–Mo–S edges [12]. This boosted surface area combined with more exposed electroactive S–Mo–S edges may contribute to the superior HER catalytic activity and enhanced supercapacitive performance.

Raman spectra were recorded to obtain structural information about the pristine MoS_2_ and the h-rGO@MoS_2_ architecture, as shown in Figs. 2c and S6. For pristine MoS_2_, the two peaks located at 371 and 401 cm^−1^ are ascribed to the E_2g_^1^ and A_g_^1^ modes, respectively [42], and the frequency difference between the E_2g_^1^ and A_g_^1^ vibrational modes is about 30 cm^−1^. Importantly, a redshift is observed after MoS_2_ nanosheets had vertically grown on h-rGO, and the frequency difference between the E_2g_^1^ and A_g_^1^ vibration modes is reduced to 27 cm^−1^, demonstrating reduced number of MoS_2_ layers [40]. This agrees well with the results obtained from the TEM images (Figs. 1g and S2). In addition, the two weak peaks situated at 1350 and 1590 cm^−1^ correspond to the D and G peaks of h-rGO, respectively [17], further revealing the successful formation of h-rGO@MoS_2_ composite with a trace amount of h-rGO.

To identify the chemical composition and surface electronic states of pristine MoS_2_ and h-rGO@MoS_2_, XPS measurements were carried out. The survey spectra of pristine MoS_2_ and h-rGO@MoS_2_ are shown in Fig. 2d and contain peaks corresponding to sulfur, molybdenum, carbon, and oxygen, and the calculated Mo/S atomic ratio is about 1/2, confirming the formation of MoS_2_. As shown in Fig. S7, the C 1*s* peaks of pristine MoS_2_ and h-rGO@MoS_2_ can be deconvoluted into three peaks and assigned to the *sp*^2^ hybrid C (C–C/C=C, 284.8 eV), hydroxyl C (C–O, 286.5 eV), and epoxy C (C=O, 288.5 eV), respectively [42]. It should be noted that the carbon in pristine MoS_2_ is mainly derived from the carbon additive used during measurement. Importantly, for the C 1*s* peaks of h-rGO@MoS_2_, the oxygen-containing groups yield low-intensity peaks, demonstrating that GO has been successfully reduced to rGO. The high-resolution Mo 3*d* spectrum of pristine MoS_2_ (Fig. 2e) can be deconvoluted into two doublet peaks located at 232.8 and 229.4 eV and at 231.8 and 228.6 eV, assigned to Mo^6+^ and Mo^4+^, respectively. In addition, the small peak at 235.5 eV probably originated from (NH_4_)_2_MoS_4_ formed during the hydrothermal reaction. Concerning the Mo 3*d* spectrum of h-rGO@MoS_2_, a single doublet peak situated at 233 and 229.8 eV can be attributed to Mo 3d_3/2_ and Mo 3d_5/2_, respectively, revealing the dominance of Mo^4+^ in MoS_2_. Notably, a positive shift in the Mo 3d spectrum is observed when forming h-rGO@MoS_2_, demonstrating the intense electron coupling effects between h-rGO and MoS_2_ [17]. Likewise, the high-resolution S 2*p* spectra of pristine MoS_2_ and h-rGO@MoS_2_ were measured, as shown in Fig. 2f. Two peaks fitted to S 2*p*_1/2_ and 2*p*_3/2_ appeared in the spectra of both MoS_2_ and h-rGO@MoS_2_, verifying the existence of terminal S^2−^ ions, which are favorable for HER activity [43]. Moreover, the unexpected peak located at 164.3 eV in the spectrum of pristine MoS_2_ is derived from (NH_4_)_2_MoS_4_, as discussed above.

**Fig. 3:**
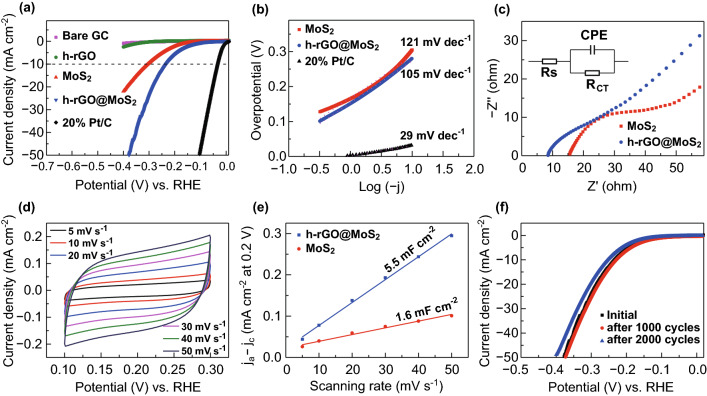
**a** Polarization curves of bare GC, h-rGO, pristine MoS_2_, h-rGO@MoS_2_, and commercial 20% Pt/C in 0.5 M H_2_SO_4_ electrolyte at 5 mV s^−1^, and **b** the corresponding Tafel plots of electrocatalysts in **a**. **c** EIS plots of pristine MoS_2_ and h-rGO@MoS_2_ from 10^−2^ to 10^5^ Hz. **d** The electrochemical double-layer capacitances of h-rGO@MoS_2_ ranging from 0.1 to 0.3 V at various scan rates (5, 10, 20, 30, 40, and 50 mV s^−1^) in 0.5 M H_2_SO_4_. **e** The capacitive current at 0.20 V as a function of scan rate for pristine MoS_2_ and h-rGO@MoS_2_ in 0.5 M H_2_SO_4_ electrolyte. **f** Polarization curves of the h-rGO@MoS_2_ electrocatalysts initially and after 1000 and 2000 cycles in 0.5 M H_2_SO_4_ electrolyte

Linear sweep voltammetry (LSV) was carried out to understand the HER performance of h-rGO@MoS_2_; bare GC, h-rGO, pristine MoS_2_, and commercial Pt/C (20%) were also evaluated as a comparison. The corresponding polarization curves in Fig. 3a were obtained at a sweep rate of 5 mV s^−1^ in 0.5 M H_2_SO_4_ without *i*R compensation. Bare GC and h-rGO exhibit negligible HER performance, even at overpotential of 400 mV. In contrast, commercial Pt/C (20%) shows the best HER performance with near-zero onset overpotential. As for h-rGO@MoS_2_, the onset potential is found at ca. 105 mV, which is much lower than that of pristine MoS_2_ (− 162 mV). Further, the overpotential of h-rGO@MoS_2_ at 10 mA cm^−2^ is about 230 mV, about 76 mV lower than that of pristine MoS_2_ (306 mV). To elucidate the HER mechanism further, Tafel slopes are extracted from the linear portions of the Tafel plots (Fig. 3b). Commercial Pt/C (20%) exhibits a Tafel slope as low as 29 mV dec^−1^, as previously reported [44]. For pristine MoS_2_ and h-rGO@MoS_2_, the Tafel slopes are calculated to be 121 and 105 mV dec^−1^, respectively, demonstrating that the Volmer–Heyrovsky mechanism is the rate-limiting step [17]. The performance of the h-rGO@MoS_2_ architecture is compared to those of other previously reported hollow MoS_2_ microspheres or rGO/MoS_2_ composites in Table S1. Because of the specific hollow architecture with vertically aligned MoS_2_ nanosheets, the h-rGO@MoS_2_ shows a superior onset overpotential.

EIS tests were carried out for both MoS_2_ and h-rGO@MoS_2_ to estimate the internal resistance properties. As shown by Fig. 3c, the charge transfer resistance of h-rGO@MoS_2_ is about 15 Ω, which is smaller than its counterpart MoS_2_ (28 Ω). This smaller internal resistance stems from the increased conductivity and shortened electron transfer pathways [33], which favor ion permeation and electron transfer, thus improving the HER catalytic performance. Besides the internal resistance, the electrochemical active surface area (ECSA) is another significant factor that has an impact on the HER performance. The corresponding electrochemical double-layer capacitances of h-rGO@MoS_2_ and MoS_2_ were measured by CV (ranging from 0.1 to 0.3 V) at different scan rates, as shown in Figs. 3d and S8. Moreover, a linear relationship between the scan rate and current density is observed, as shown in Fig. 3e. Based on the calculation, h-rGO@MoS_2_ has a capacitance of 5.5 mF cm^−2^, which is 3.5 times higher than that of pristine MoS_2_. In other words, the electrochemically active sites are boosted after the vertical growth of MoS_2_ on h-rGO, and this increase in electrochemically active sites no doubt enhanced the intrinsic HER activity of MoS_2_. Additionally, long-term cycling tests of h-rGO@MoS_2_ are examined by continuous cyclic voltammetry measurements between -0.4 and 0.1 V versus RHE at 50 mV s^−1^ in Fig. 3f. Clearly, negligible performance decay is observed after the first 1000 CV cycles. Moreover, slight decay is seen after 2000 CV cycles, demonstrating the remarkable stability of h-rGO@MoS_2_.

In this study, hollow rGO spheres were used as a matrix to confine the growth of MoS_2_ and prevent its agglomeration. Note that the improved HER catalytic activity is derived from a synergistic effect, combining the following three aspects: (1) the expanded (002) interlayer spacing that increases the diffusion kinetics of ions and the intralayer conductivity [41]; (2) the ultrathin vertically aligned MoS_2_ with a large number of exposed electroactive sites and increased contact area with the electrolyte [24]; and (3) the improved conductivity that accelerates electron transfer [45]. Thanks to the aforementioned synergistic effects, h-rGO@MoS_2_ shows superior HER performance.

**Fig. 4:**
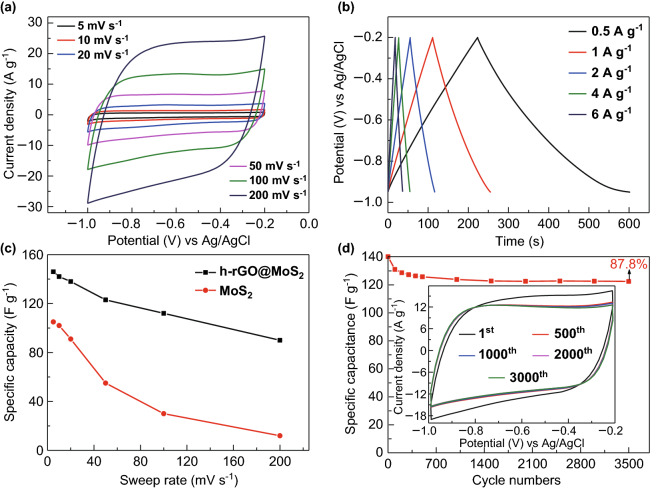
**a** CV curves of h-rGO@MoS_2_ electrode at various sweep rates (5, 10, 20, 50, 100, and 200 mV s^−1^) in 1 M Na_2_SO_4_ electrolyte. **b** Galvanostatic curves of h-rGO@MoS_2_ at various current densities (0.5, 1, 2, 4, and 6 A g^−1^). **c** Capacitance retention property of h-rGO@MoS_2_ and MoS_2_ at sweep rates from 5 to 200 mV s^−1^. **d** Cycle stability and capacitance retention of the h-rGO@MoS_2_ electrode tested at a scan rate of 100 mV s^−1^; the inset shows the CV curves for the 1st, 500th, 1000th, 2000th, and 3000th cycles

Further, the supercapacitive performance of h-rGO@MoS_2_ was evaluated using CV and GCD measurements in 1 M Na_2_SO_4_ electrolyte. As shown in Fig. 4a, various applied scan rates (ranging from 5 to 200 mV s^−1^) were selected to evaluate the rate performance of h-rGO@MoS_2_. With increasing scan rate, the current densities gradually increased, and they remain almost near-rectangular at all tested scan rates, suggesting the high rate performance of h-rGO@MoS_2_. The specific capacitance is calculated as *C*_s_ = ∫*I*d*v*/*vm*Δ*V*, where *I* (A), *v* (V s^−1^), Δ*V* (V), and *m* (g) are the response current, scan rate, potential window, and the mass of active electrode material, respectively. According to the CV curves, the specific capacitances are found to be 146, 142, 138, 123, 112, and 90 F g^−1^ at scan rates of 5, 10, 20, 50, 100, and 200 mV s^−1^, respectively. In the case of the pristine MoS_2_ electrode, however, the shapes of the CV curves change from near-rectangular to twisted ellipses as the scan rate increased from 5 to 200 mV s^−1^, revealing its less capacitive but more resistive performance (Fig. S9). To compare the capacitance and rate performance of MoS_2_ and the h-rGO@MoS_2_ architecture, the CV curves are compared at a sweep rate of 100 mV s^−1^ in Fig. S10. The CV curve of the h-rGO@MoS_2_ architecture has a typical rectangular shape with a large area, while the shape of pristine MoS_2_ has become a tilted ellipse.

Regarding the specific capacitance, h-rGO@MoS_2_ shows a higher capacitance value (112 F g^−1^) than that of pristine MoS_2_ (30 F g^−1^). This high capacitance and good rate performance arise from the formation of vertical 3D architectures, as shown in Fig. 1d, which allow electrolyte ions to access the interior surfaces of the electrode more easily and shorten the permeation distance, resulting in increased capacitive performance. Galvanostatic charge–discharge measurements were conducted at various current densities from 0.5 to 6 A g^−1^ in a potential window of − 1 to − 0.2 V. As shown in Fig. 4b, the charge curves are approximately linear and symmetric with their discharge counterparts, which further indicate the double electrode layer capacitance and excellent reversibility of the h-rGO@MoS_2_ architecture [16]. The specific capacitances of the h-rGO@MoS_2_ architecture are calculated using *C *=* I*Δ*t/*Δ*V*, where *C* is the specific capacitance, *I* is the constant discharge current density, Δ*t* is the discharging time, and Δ*V* is the potential window. The specific capacitance of h-rGO@MoS_2_ was calculated to be 238 F g^−1^ at a current density of 0.5 A g^−1^.

Moreover, even at a high current density of 6 A g^−1^ the specific capacitance remained 135 F g^−1^, which demonstrates the excellent rate performance. However, the specific capacitance of pristine MoS_2_ can only reach 106 F g^−1^ at a current density of 0.5 A g^−1^, and reduced to 41.2 F g^−1^ when the current density is increased to 6 A g^−1^ (Fig. S11). The supercapacitive performances of MoS_2_/graphene-based materials are summarized and compared in Table S2. As shown, the h-rGO@MoS_2_ architecture has a better specific capacitance than the other materials, which probably originates from the synergistic effect of the large surface area for the adsorption–desorption of ions and increased conductivity.

The capacitance during ultrafast charging/discharging was tested for pristine MoS_2_ and h-rGO@MoS_2_ (the results are shown in Fig. 4c). At a sweep rate of 5 mV s^−1^, the specific capacitance of h-rGO@MoS_2_ is ca. 146 F g^−1^, which is ca. 1.4 times higher than that of pristine MoS_2_ (105 F g^−1^). As the sweep rate increased from 5 to 200 mV s^−1^, the specific capacitance of the h-rGO@MoS_2_ electrode decreased, but still achieved remarkable capacitance retention of 60% with a 40-fold increase in sweep rate. The specific capacitance of pristine MoS_2_ decreased sharply with increasing sweep rate and only 10% capacitance is retained at sweep rate of 200 mV s^−1^. The significant discrepancy in the rate capabilities of the h-rGO@MoS_2_ electrode and pristine MoS_2_ demonstrate that the introduction of graphene and the formation of vertical architectures contributed to boosting the capacitance retention performance.

The cycling stability is another significant factor that impacts the large-scale application of supercapacitors. The evaluation of the cycling stability of pristine MoS_2_ and h-rGO@MoS_2_ was investigated by CV cycling at a sweep rate of 100 mV s^−1^. As shown in Fig. S12, pristine MoS_2_ displayed stable cyclic behavior. With the 3D vertical architecture, the capacitance of the h-rGO@MoS_2_ electrode showed an approximately 10% reduction in the first 400 cycles (Fig. 4d), which probably arises from the volume change of the active materials during the CV processes. In the subsequent 500–3500 cycles, the capacitance of h-rGO@MoS_2_ remained steady at 87.7% of its initial capacitance, demonstrating the excellent long-term cycling stability.

## Conclusions

In summary, 3D rGO hollow sphere-supported ultrathin MoS_2_ nanosheets have been prepared, and show enhanced HER catalytic activity and supercapacitive performance compared to pristine MoS_2_. The enhanced HER catalytic activity and supercapacitive performance are mainly derived from the following three factors: (1) the vertically aligned hierarchical architecture, which provides a large surface area for the adsorption, desorption, and diffusion of ions; (2) the relatively thin MoS_2_ sheets, which provides more active sites and expanded interlayer spacing; and (3) the increased conductivity. Our strategy for constructing 3D architectures of vertically aligned nanosheets supported on hollow spheres may be applied to 3D composites of other materials and offers the potential for the development of efficient HER catalysts and advanced supercapacitor electrodes.

## Electronic supplementary material

Below is the link to the electronic supplementary material.

Supplementary material 1 (PDF 841 kb)
