# Supplementary material for: MoS2 Nanosheet Arrays Rooted on Hollow rGO Spheres as Bifunctional Hydrogen Evolution Catalyst and Supercapacitor Electrode
Source: Nanomicro Lett. 2018 Jul 9;10(4):62. doi: 10.1007/s40820-018-0215-3 (PMC6199108; doi:10.1007/s40820-018-0215-3)
Supplement: Supplementary file 1 — Supplementary material 1 (PDF 841 kb) [file 40820_2018_215_MOESM1_ESM.pdf]

Supporting Information for

MoS_2 Nanosheets Arrays Rooted on Hollow rGO Spheres as Bifunctional Hydrogen Evolution Catalyst and Supercapacitor Electrode

Shizheng Zheng[1], Lijun Zheng[1], Zhengyou Zhu[1], Jian Chen[1], Jianli Kang[2], Zhulin Huang[3], Dachi Yang[1,*]

[1]Key Laboratory of Photoelectronic Thin Film Devices and Technology of Tianjin; Department of Electronics, College of Electronic Information and Optical Engineering, Nankai University, Tianjin 300350, People's Republic of China

[2]School of Material Science and Engineering, Tianjin Polytechnic University, Tianjin 300387, People's Republic of China

[3]Key Laboratory of Materials Physics, and Anhui Key Laboratory of Nanomaterials and Nanotechnology, Institute of Solid State Physics, Chinese Academy of Sciences, Hefei 230031, People's Republic of China

*Corresponding author. E-mail: yangdachi@nankai.edu.cn (Dachi Yang)

Supplementary Figures and Tables

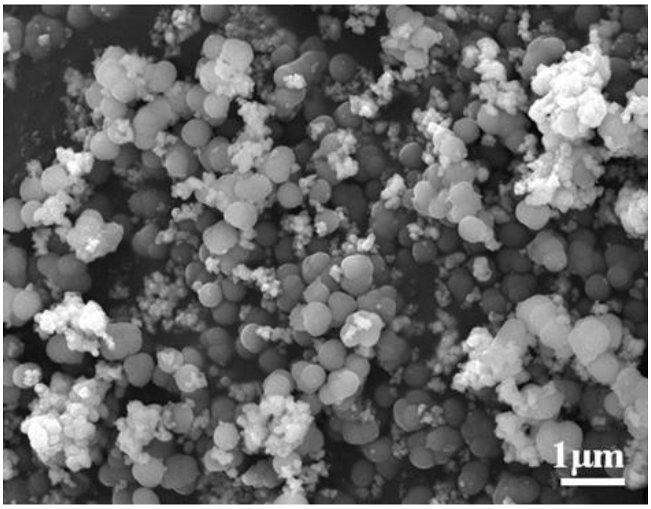

Fig. S1 SEM image of SiO_2/MoS_2 composites

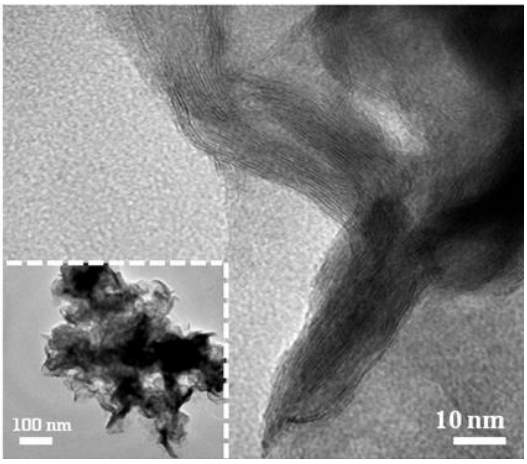

Fig. S2 TEM images of pristine MoS_2. The inset is the low magnification TEM image

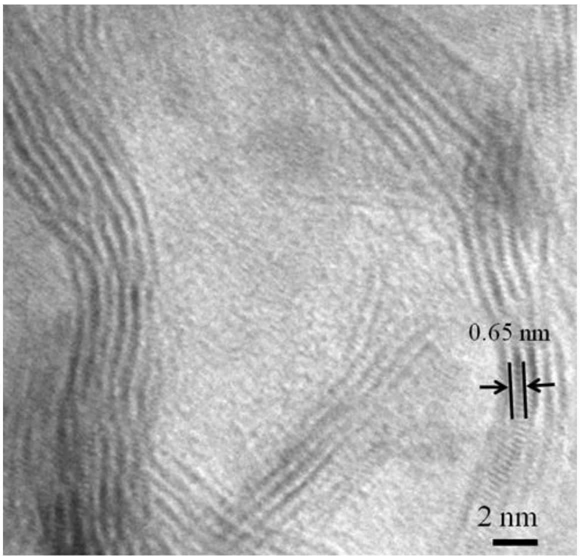

Fig. S3 HRTEM of h-rGO@MoS_2 with expanded (002) interlayer spacing

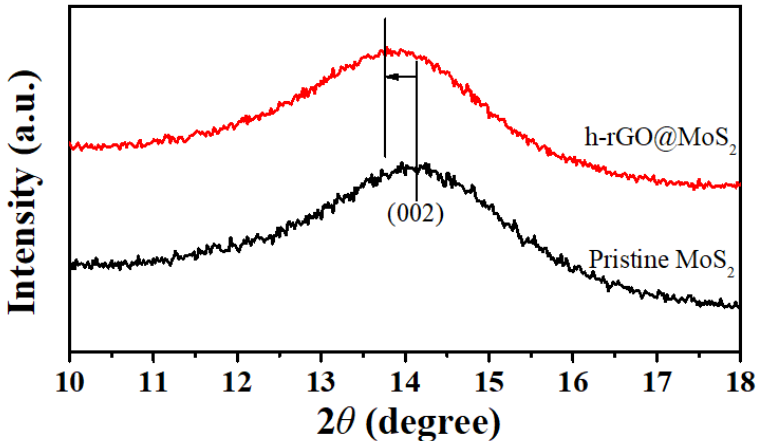

Fig. S4 XRD patterns of the (002) plane peaks of pristine MoS_2 and h-rGO@MoS_2

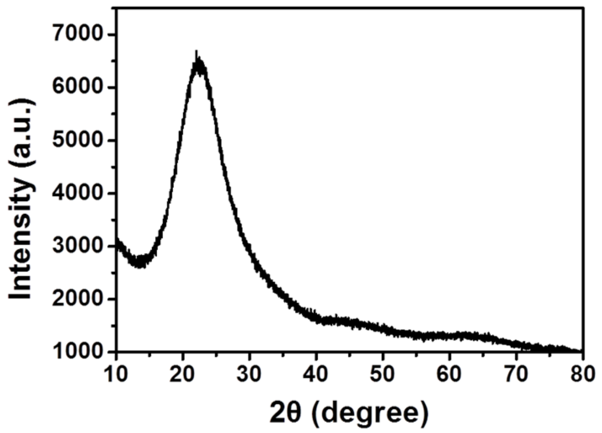

Fig. S5 XRD patterns of amorphous SiO_2 template

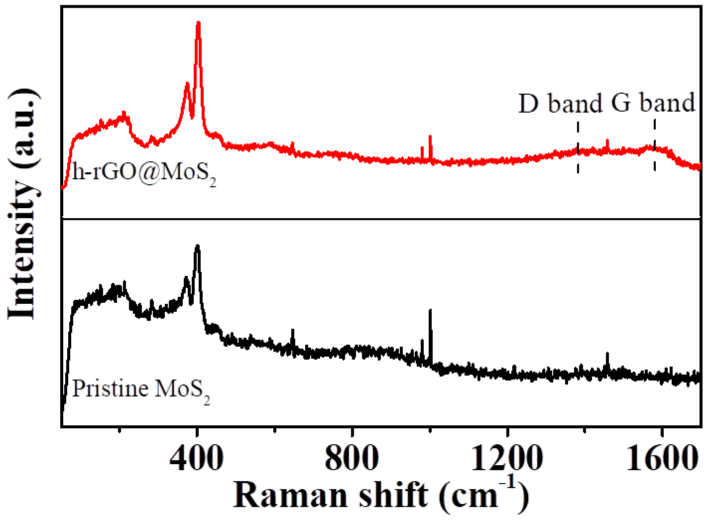

Fig. S6 Raman spectra of pristine MoS_2 and h-rGO@MoS_2

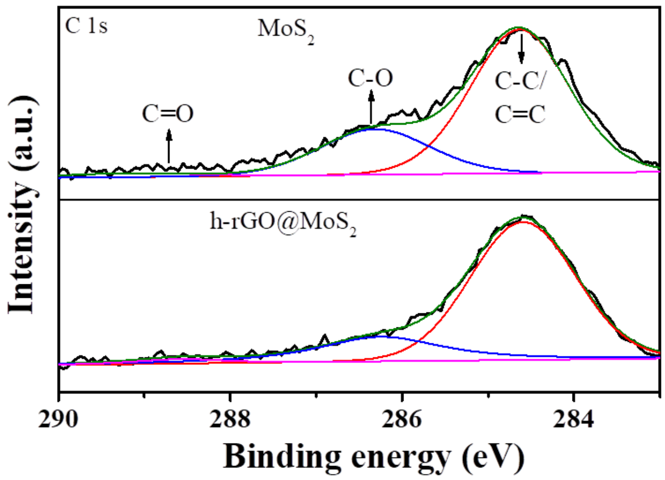

Fig. S7 The high resolution C 1s XPS spectra of pristine MoS_2 and h-rGO@MoS_2

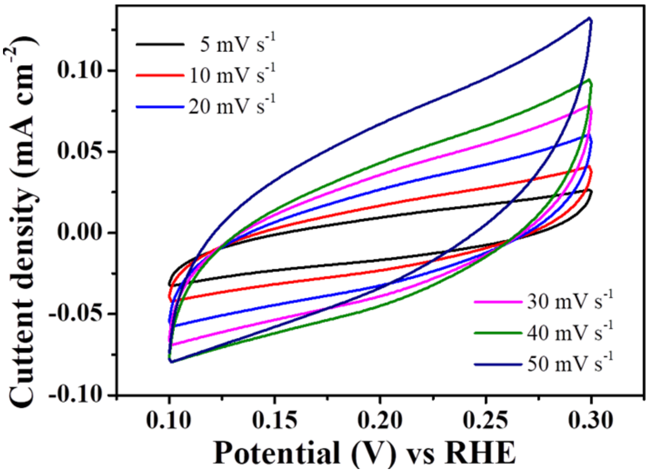

Fig. S8 Electrochemical double-layer capacitances of pristine MoS_2 ranged from 0.1 to 0.3 V at various scan rates (5, 10, 20, 30, 40, and 50 mV s^{-1})

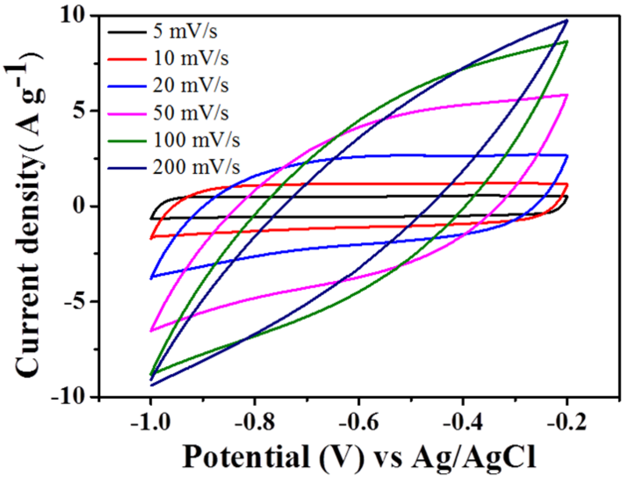

Fig. S9 CV curves of pristine MoS_2 at different sweep rates

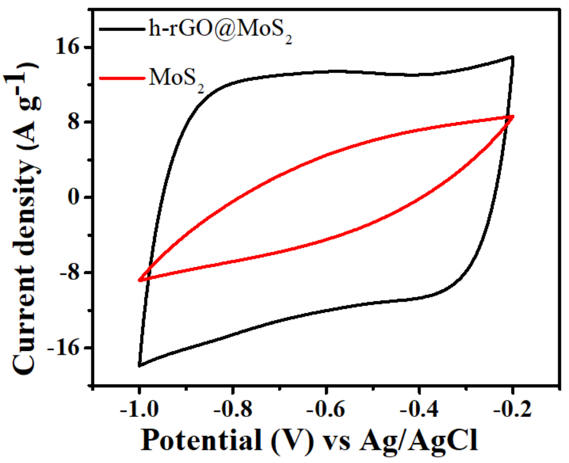

Fig. S10 CV curves contrast between h-rGO@MoS_2 and pristine MoS_2 at scan rate of 100 mV s^{-1}

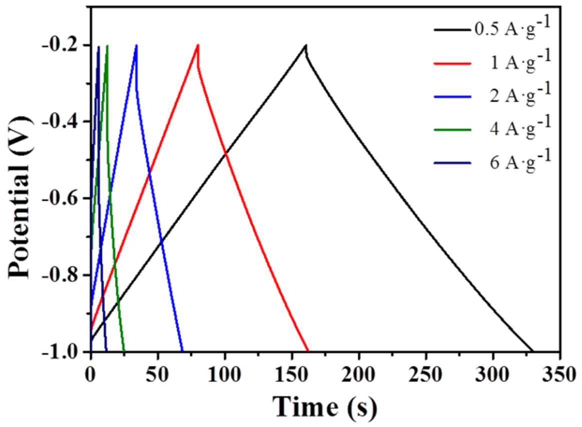

Fig. S11 GCD curves of pristine MoS_2 at various current densities

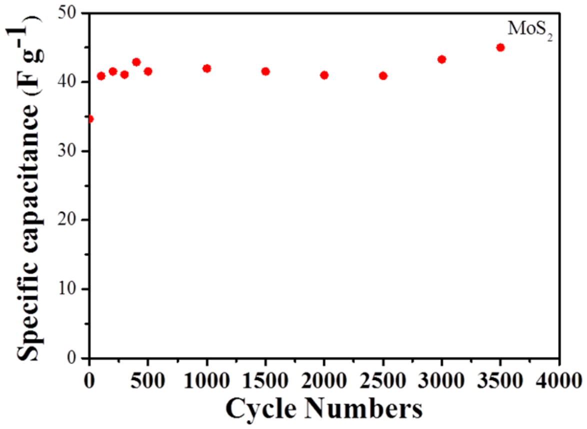

Fig. S12 3500 loops cycle stability of MoS_2 at scan rate of 100 mV s^{-1}

Table S1 MoS_2/graphene electrocatalysts and their performance comparison in onset overpotential and Tafel slope

Electrocatalysts	Onset overpotential (mV)	Tafel slope (mV/decade)	Ref.
(0D/3D) MoS_2 on porous graphene	150	56	1
MoS_2/GO hybrid	150	91.9	2
MoS_2/N-doped graphene nanosheet Aerogels	236	230	3
Microwave-assisted synthesized MoS_2/ graphene	170	80	4
MoS_2 nanoflower-decorated rGO paper	190	90	5
3D MoS_2/rGO hierarchical frameworks	121	46.5	6
MoS_2 vertically rooted on hollow rGO	105	105	This work

Table S2 MoS_2/graphene electrode materials and their capacitive performance in various electrolytes

Electrode material	Specific capacitance ($F\ g^{-1}$)	Current density ($A\ g^{-1}$)	Electrolyte	Ref.
hollow MoS_2	142	1.18	1 M KCl	7
Layered MoS_2-graphene	243	1	1M Na_2SO_4	8
MoS_2/RGO	216	1	1 M H_2SO_4	9
MoS_2/N-doped graphene	245	0.25	1M KOH	10
Assembled MoS_2 microflower	167.7	1	3M KOH	11
h-rGO@MoS_2	238	0.5	1 M Na_2SO_4	This work

Reference

[1] Y. Liu, Y. Zhu, X. Fan, S. Wang, Y. Li, F. Zhang, G. Zhang, W. Peng, (0D/3D) MoS_2 on porous graphene as catalysts for enhanced electrochemical hydrogen evolution. Carbon **121**, 163-169 (2017). https://doi.org/10.1016/j.carbon.2017.05.092

[2] E.G. Firmiano, M.A. Cordeiro, A.C. Rabelo, C.J. Dalmaschio, A.N. Pinheiro, E.C. Pereira, E.R. Leite, Graphene oxide as a highly selective substrate to synthesize a layered MoS_2 hybrid electrocatalyst. Chem. Commun. **48**(62), 7687-7689 (2012). https://doi.org/10.1039/c2cc33397j

[3] Y. Hou, B. Zhang, Z. Wen, S. Cui, X. Guo, Z. He, J. Chen, A 3D hybrid of layered MoS_2/nitrogen-doped graphene nanosheet aerogels: An effective catalyst for hydrogen evolution in microbial electrolysis cells. J. Mater. Chem. A **2**(34), 13795-13800 (2014). https://doi.org/10.1039/c4ta02254h

[4] J. Cao, X. Zhang, Y. Zhang, J. Zhou, Y. Chen, X. Liu, Free MoS_2 nanoflowers grown on graphene by microwave-assisted synthesis as highly efficient non-noble-metal electrocatalysts for the hydrogen evolution reaction. PLoS One **11**(8), e0161374 (2016). https://doi.org/10.1371/journal.pone.0161374

[5] C.B. Ma, X. Qi, B. Chen, S. Bao, Z. Yin et al., MoS_2 nanoflower-decorated reduced graphene oxide paper for high-performance hydrogen evolution reaction. Nanoscale **6**(11), 5624-5629 (2014). https://doi.org/10.1039/c3nr04975b

[6] W. Zhou, K. Zhou, D. Hou, X. Liu, G. Li, Y. Sang, H. Liu, L. Li, S. Chen, Three-dimensional hierarchical frameworks based on MoS_2 nanosheets self-assembled on graphene oxide for efficient electrocatalytic hydrogen evolution. ACS. Appl. Mater. Interfaces **6**(23), 21534-21540 (2014). https://doi.org/10.1021/am506545g

[7] L. Wang, Y. Ma, M. Yang, Y. Qi, Hierarchical hollow MoS_2 nanospheres with enhanced electrochemical properties used as an electrode in supercapacitor. Electrochim. Acta **186**, 391-396 (2015). https://doi.org/10.1016/j.electacta.2015.10.130

[8] K.-J. Huang, L. Wang, Y.-J. Liu, Y.-M. Liu, H.-B. Wang, T. Gan, L.-L. Wang, Layered MoS_2–graphene composites for supercapacitor applications with enhanced capacitive performance. Int. J. Hydrog. Energy **38**(32), 14027-14034 (2013). https://doi.org/10.1016/j.ijhydene.2013.08.112

[9] X. Li, C. Zhang, S. Xin, Z. Yang, Y. Li, D. Zhang, P. Yao, Facile synthesis of MoS_2/reduced graphene oxide@polyaniline for high-performance supercapacitors. ACS Appl. Mater. Interfaces **8**(33), 21373-21380 (2016). https://doi.org/10.1021/acsami.6b06762

[10] B. Xie, Y. Chen, M. Yu, T. Sun, L. Lu, T. Xie, Y. Zhang, Y. Wu, Hydrothermal synthesis of layered molybdenum sulfide/N-doped graphene hybrid with enhanced supercapacitor performance. Carbon **99**, 35-42 (2016). https://doi.org/10.1016/j.carbon.2015.11.077

[11] L.-M. Xu, L. Ma, X.-Y. Xu, X.-P. Zhou, L.-L. Zhang, W.-X. Chen, Molybdenum disulfide microflowers assembled by few-layered nanosheets and their electrochemical performance for supercapacitor. Mater. Lett. **173**, 84-87 (2016). https://doi.org/10.1016/j.matlet.2016.03.027
